# Hemiarthroplasty Versus Total Hip Arthroplasty for Femoral Neck Fracture in Elderly Patients: Twelve-Month Risk of Revision and Dislocation in an Instrumental Variable Analysis of Medicare Data

**DOI:** 10.2106/JBJS.23.00247

**Published:** 2023-09-06

**Authors:** Adam I. Edelstein, Timothy R. Dillingham, Emily L. McGinley, Liliana E. Pezzin

**Affiliations:** 1 Department of Orthopaedic Surgery, Northwestern University Feinberg School of Medicine, Chicago, Illinois; 2 Department of Physical Medicine and Rehabilitation, University of Pennsylvania, Philadelphia, Pennsylvania; 3 Center for Advancing Population Science, Medical College of Wisconsin, Milwaukee, Wisconsin; 4 Institute for Health and Equity, Medical College of Wisconsin, Milwaukee, Wisconsin

## Abstract

**Background:**

There is practice variation in the selection of a total hip arthroplasty (THA) or a hemiarthroplasty (HA) for the treatment of displaced femoral neck fractures in elderly patients. Large data sets are needed to compare the rates of rare complications following these procedures. We sought to examine the relationship between surgery type and secondary hip surgery (revision or conversion arthroplasty) at 12 months following the index arthroplasty, and that between surgery type and dislocation at 12 months, among elderly Medicare beneficiaries who underwent THA or HA for a femoral neck fracture, taking into account the potential for selection bias.

**Methods:**

We performed a population-based, retrospective study of elderly (>65 years of age) Medicare beneficiaries who underwent THA or HA following a femoral neck fracture. Two-stage, instrumental variable regression models were applied to nationally representative Medicare medical claims data from 2017 to 2019.

**Results:**

Of the 61,695 elderly patients who met the inclusion criteria, of whom 74.1% were female and 92.2% were non-Hispanic White, 10,268 patients (16.6%) underwent THA and 51,427 (83.4%) underwent HA. The findings from the multivariable, instrumental variable analyses indicated that treatment of displaced femoral neck fractures with THA was associated with a significantly higher risk of dislocation at 12 months compared with treatment with HA (2.9% for the THA group versus 1.9% for the HA group; p = 0.001). There was no significant difference in the likelihood of 12-month revision/conversion between THA and HA.

**Conclusions:**

The use of THA to treat femoral neck fractures in elderly patients is associated with a significantly higher risk of 12-month dislocation, as compared with the use of HA, although the difference may not be clinically important. A low overall rate of dislocation was found in both groups. The risk of revision/conversion at 12 months did not differ between the groups.

**Level of Evidence:**

Therapeutic Level III. See Instructions for Authors for a complete description of levels of evidence.

Femoral neck fractures represent a major source of morbidity for elderly individuals. By 2050, the global annual incidence is expected to rise to between 7 and 21 million cases owing to an aging population and increased life expectancy^1^. Hip arthroplasty in the form of hemiarthroplasty (HA) or total hip arthroplasty (THA) for the treatment of displaced femoral neck fractures in elderly patients has been established as the standard of care and has been shown to enable rapid mobilization and satisfactory long-term outcomes^2-4^.

There is practice variation in the selection of THA versus HA for the treatment of displaced femoral neck fracture in elderly patients^5-7^. Proponents of THA cite evidence of better outcomes associated with THA as compared with HA, such as improved function, quality of life, and implant survival, which have been demonstrated in several randomized controlled trials (RCTs)^8-13^. Advocates of HA point to the association of THA with longer operative time, higher blood loss, and a higher risk of dislocation, without clear evidence of clinically meaningful improved function or implant survival, as has been shown in other RCTs^14-17^. Recently, a large, international randomized trial demonstrated no significant differences between THA and HA in the rates of secondary hip procedures at 2 years, although a nonsignificant trend toward higher rates of early revision surgery was identified in the THA group^16^.

Given the rarity of certain adverse events such as dislocation and early revision, there may be differences in outcomes between THA and HA that are not easily detected by randomized trials. To detect differences in these rare events, an analysis of large data sets may be helpful. Several recent population-based analyses from outside of the U.S. have investigated the risk of these early adverse events, with conflicting results^18,19^. To our knowledge, no studies of nationally representative data sets that correct for selection bias have been performed on THA versus HA outcomes following femoral neck fractures in the U.S. The purpose of this study was to leverage a large, national data set in order to evaluate the relationship of surgery type, specifically THA versus HA, to the rate of revision or conversion arthroplasties and to the rate of dislocations at 12 months postoperatively among elderly persons undergoing arthroplasty for a displaced femoral neck fracture. We hypothesized that THA would be associated with higher rates of dislocation and revision at 12 months.

## Materials and Methods

### Study Population and Data Sources

Medicare medical claims data from the Centers for Medicare & Medicaid Services were utilized to identify all fee-for-service beneficiaries with a femoral neck fracture treated with THA or HA during 2017 and 2018. THAs and HAs were identified with use of the International Classification of Diseases, 10th Revision, Clinical Modification (ICD-10-CM) procedure codes. Femoral neck fractures were identified with use of the following ICD-10-CM diagnostic codes: S72.00XX, S72.01XX, S72.02XX, S72.03XX, S72.04XX, and S72.09XX. To calculate comorbidity, patients were required to have been enrolled in fee-for-service Medicare for ≥12 months prior to surgery. We excluded patients who had arthroplasty for a fracture related to an oncologic or infectious process and those who were undergoing revision or conversion procedures.

### Outcome Measures

The key outcomes for this study were secondary hip surgery (revision or conversion arthroplasty) at 12 months following the index arthroplasty and dislocation at 12 months following the index arthroplasty. Patients with THA were classified as undergoing a revision if they had an ICD-10-CM revision code or codes for both implant removal and replacement with laterality matching that of the index arthroplasty, as previously described^20^. Patients with HA were classified as undergoing revision/conversion if they had a code for revision of the femur, codes for removal and replacement of the femur, or a code for replacement of the acetabulum, again with laterality matching that of the index HA^20^. Similar to previously published methodology^21^, patients were classified as having a dislocation if there were any of the following ICD-10-CM dislocation codes during the 12 months following the index hip surgery, with matching laterality: T84.02XX and S73.0XXX.

### Surgery Type

The primary independent variable of interest was the type of surgery, classified as either THA or HA. Data related to the surgical approach were not available in the Medicare data set and were not included in the analyses. Patients were categorized as having undergone a THA if they had the ICD-10-CM procedure codes 0SR90J9, 0SR90JA, 0SR90JZ, 0SRB0J9, 0SRB0JA, 0SRB0JZ, 0SR9019, 0SR901A, 0SR901Z, 0SR9029, 0SR902A, 0SR902Z, 0SR9039, 0SR903A, 0SR903Z, 0SR9049, 0SR904A, 0SR904Z, 0SRB019, 0SRB01A, 0SRB01Z, 0SRB029, 0SRB02A, 0SRB02Z, 0SRB039, 0SRB03A, 0SRB03Z, 0SRB049, 0SRB04A, or 0SRB04Z. Patients were identified as having undergone an HA if they had the ICD-10-CM procedure codes 0SRR019, 0SRR01A, 0SRR01Z, 0SRR039, 0SRR03A, 0SRR03Z, 0SRR0J9, 0SRR0JA, 0SRR0JZ, 0SRS019, 0SRS01A, 0SRS01Z, 0SRS039, 0SRS03A, 0SRS03Z, 0SRS0J9, 0SRS0JA, or 0SRS0JZ.

### Other Covariates

In addition to surgery type, all analyses were adjusted for patient age, sex, race/ethnicity (as listed in the Medicare data set), and low-income status, the latter of which was proxied by dual enrollment in Medicare and in either Medicaid or a state buy-in program. We also included controls for the number of comorbidities, based on Medicare data for the 12 months preceding the index arthroplasty in accordance with the Elixhauser algorithm^22,23^, and the U.S. Census Bureau region of the hospital. We included femoral component cement status as a covariate, which was classified as cemented, uncemented, or unspecified on the basis of ICD-10 codes^24^.

### Statistical Analysis

Our primary goal was to estimate the relationship between surgery type (THA versus HA) and outcomes (revision/conversion and dislocation) at 12 months postoperatively, controlling for potential confounders. One important econometric issue complicated the estimation process: the selection bias regarding the nonrandom “assignment” to surgery type based on differential surgeon-patient decision-making related to variables unobserved in the data set. For example, bone quality, which was unobserved in the data set, might have simultaneously affected the choice of THA versus HA and the probability (conditional on the type of surgery) of adverse outcomes.

We applied the leading statistical method for addressing this type of bias: the 2-stage instrumental variable technique^25-28^. In the first stage of the analysis, the likelihood that a patient would undergo either a THA or HA was estimated with use of a probit specification. Residuals from this first stage, along with surgery type, sociodemographics, and clinical factors included in the first-stage analysis, were included as additional regressors in the revision/conversion and dislocation equations during the second-stage analysis. The resulting estimates of the effect of surgery type on outcomes would be less affected by selection bias.

Instrumental variables are effective only if valid “instruments” are available. Specifically, instrumental variables must be predictive of the type of surgery that a patient underwent but not independently predictive of surgical outcomes, which are conditional on the type of surgery performed. Guided by prior research, we identified 3 instrumental variables as potentially important determinants of a surgeon’s choice of surgery: (1) the annual overall volume of THAs (elective procedures and those performed for fractures) at the surgeon’s practicing facility, (2) the annual proportion of THAs at the surgeon’s practicing facility that were performed for fracture, and (3) the annual proportion of femoral neck fracture cases at the surgeon’s practicing facility that were treated with HA^25^. All volume variables were averaged over a 2-year period.

We examined the validity of our proposed instruments with use of the Stock and Staiger test, which was based on the partial r^2^ and Chow F statistics for the excluded variables in the first-stage regression analysis^26^. We tested the adequacy of the instruments with respect to whether they could be legitimately excluded from the second-stage outcome estimations that included surgery type and first-stage residuals^27^. In all analyses, standard errors were adjusted to account for potential clustering (i.e., multiple patients within the same hospital).

To provide a sense of the magnitude of the effects, we calculated the predicted probabilities (i.e., adjusted risks) for key variables by varying a specific characteristic (e.g., patient’s race, low-income status) while holding all other variables constant at their original levels. These predicted probabilities were calculated at the individual level and then averaged over the entire sample.

All statistical analyses were performed using Stata 16 (StataCorp). The level of significance was set at p < 0.05.

### Source of Funding

This work was supported by the National Institutes of Health grant 5-R01-AG058718. Grant funds were utilized to obtain the Medicare data and to support the time of our statistician.

## Results

We identified a total of 62,489 elderly (66 to 93 years of age) Fee-for-Service (FFS) Medicare beneficiaries who underwent arthroplasty for a femoral neck fracture during 2017 to 2018 who (1) were enrolled in fee-for-service Medicare at least 12 months prior to their index surgery and (2) were alive and continuously enrolled during the 12-month follow-up period after the index arthroplasty. We excluded 445 patients for unspecified surgery type, 262 patients for missing race/ethnicity or dual enrollment status, and 87 patients for missing Census region. The final sample comprised 61,695 patients, of whom 10,268 (16.6%) received THA and 51,427 (83.4%) received HA.

Patient characteristics are shown in Table I. The HA group was significantly older: 68.5% of patients in the HA group were ≥80 years of age versus 40.5% of patients in the THA group. Compared with patients in the THA group, those in the HA group were more likely to be female (74.5% versus 71.8%; p < 0.001), to be of a minority race/ethnicity (8.1% versus 6.4%; p < 0.001), to have a higher number of comorbid conditions (mean and standard deviation [SD], 3.4 ± 2.8 versus 2.8 ± 2.6; p < 0.001), and to have low income (Medicaid dual enrollment, 13.7% versus 7.7%; p < 0.001).

**TABLE I:** Characteristics of the Cohort, Overall and by Surgery Type

	Overall (N = 61,695)	THA (N = 10,268)	HA (N = 51,427)	P Value
Age, in years *(no. [%] of patients)*				<0.001
66-69	3,486 (5.7%)	1,427 (13.9%)	2,059 (4.0%)	
70-74	7,457 (12.1%)	2,284 (22.2%)	5,173 (10.1%)	
75-79	11,363 (18.4%)	2,394 (23.3%)	8,969 (17.4%)	
80-84	15,016 (24.3%)	2,090 (20.4%)	12,926 (25.1%)	
85-89	15,605 (25.3%)	1,487 (14.5%)	14,118 (27.5%)	
≥90	8,768 (14.2%)	586 (5.7%)	8,182 (15.9%)	
Sex *(no. [%] of patients)*				<0.001
Female	45,707 (74.1%)	7,372 (71.8%)	38,335 (74.5%)	
Male	15,988 (25.9%)	2,896 (28.2%)	13,092 (25.5%)	
Race/ethnicity *(no. [%] of patients)*				<0.001
Non-Hispanic White	56,860 (92.2%)	9,612 (93.6%)	47,248 (91.9%)	
Black/African American	1,632 (2.6%)	214 (2.1%)	1,418 (2.8%)	
Hispanic	1,715 (2.8%)	230 (2.2%)	1,485 (2.9%)	
Other	1,488 (2.4%)	212 (2.1%)	1,276 (2.5%)	
No. of comorbidities*	3.3 ± 2.8	2.8 ± 2.6	3.4 ± 2.8	<0.001
Low-income status *(no. [%] of patients)*				<0.001
No	53,834 (87.3%)	9,477 (92.3%)	44,357 (86.3%)	
Yes	7,861 (12.7%)	791 (7.7%)	7,070 (13.7%)	
Census region of facility *(no. [%] of patients)*				0.004
Northeast	9,887 (16.0%)	1,646 (16.0%)	8,241 (16.0%)	
South	27,418 (44.4%)	4,536 (44.2%)	22,882 (44.5%)	
Midwest	13,548 (22.0%)	2,148 (20.9%)	11,400 (22.2%)	
West	10,842 (17.6%)	1,938 (18.9%)	8,904 (17.3%)	
Use of cementation *(no. [%] of patients)*				<0.001
No	23,662 (38.4%)	4,848 (47.2%)	18,814 (36.6%)	
Yes	18,742 (30.4%)	1,793 (17.5%)	16,949 (33.0%)	
Unspecified	19,291 (31.3%)	3,627 (35.3%)	15,664 (30.5%)	
Overall facility volume of THAs*	126.7 ± 114.2	149.1 ± 136.2	122.2 ± 108.7	<0.001
Proportion of facility’s THAs performed for fractures*	0.35 ± 0.31	0.30 ± 0.20	0.41 ± 0.21	<0.001
Proportion of femoral neck fracture cases treated with HA*	0.82 ± 0.11	0.72 ± 0.22	0.91 ± 0.13	<0.001

* Values given as the mean and standard deviation.

### Factors Associated with Surgery Type

Our findings from the multivariable probit model, presented in Table II, indicate that older patients (relative to patients aged 66 to 69 years; the coefficient ranged from −0.23 for patients 70 to 74 years to −1.36 for patients ≥90 years; p < 0.001), Black patients (−0.15; p = 0.001), patients with low income (−0.33; p < 0.001), and patients with a higher comorbidity burden (−0.04; p < 0.001) were significantly less likely to undergo THA. In contrast, male patients (0.06; p < 0.001) were more likely than female patients to undergo THA.

**TABLE II:** Multivariable Regression Coefficient Estimates for Factors Associated with the Choice of THA as the Surgery Type*

	Coefficient	Standard Error	P Value
Age, in years			
66-69	Ref.	—	—
70-74	−0.23	0.028	<0.001
75-79	−0.60	0.027	<0.001
80-84	−0.91	0.027	<0.001
85-89	−1.15	0.028	<0.001
≥90	−1.36	0.033	<0.001
Sex			
Female	Ref.	—	—
Male	0.06	0.015	<0.001
Race/ethnicity			
Non-Hispanic White	Ref.	—	—
Black/African American	−0.15	0.047	0.001
Hispanic	0.008	0.044	0.84
Other	−0.05	0.047	0.31
No. of comorbidities	−0.04	0.003	<0.001
Low-income status			
No	Ref.	—	—
Yes	−0.33	0.024	<0.001
Census region of surgical facility			
Northeast	Ref.	—	—
South	−0.05	0.02	0.02
Midwest	−0.02	0.02	0.30
West	−0.08	0.02	0.001
Candidate instrumental variables			
Overall facility volume of THAs	0.0002	0.00007	0.002
Proportion of facility’s THAs performed for fracture	−0.12	0.043	0.006
Proportion of femoral neck fracture cases treated with HA	−4.03	0.054	<0.001

* The dependent variable is a binary indicator given the value of 1 if the patient received a THA and 0 if the patient received HA. The model was estimated with use of a probabilistic probit specification to account for the nonlinear, discrete nature of the dependent variable. The estimation procedure included a constant term and accounted for clustering (i.e., multiple observations within the same facility). The regression coefficients represent the adjusted marginal effect associated with a unit change in the indicator covariate, controlling for all other factors. Positive or negative coefficients indicate a higher or lower likelihood, respectively, of patients receiving THA relative to HA. Given the nonlinearity of the dependent variable, the probit regression coefficients do not represent the magnitude of the effect. To provide a sense of the magnitude of the effect for key factors, we utilized these regression coefficients to calculate the predicted (adjusted) probability of receiving a THA (relative to HA) associated with a 1-unit change in the indicator variable (e.g., Black/African American race), while holding all other factors constant at their actual values. These predicted probabilities are presented in the text.

The predicted probabilities indicated that the likelihood of undergoing THA was 38% for patients aged 66 to 69 years, 29.3% for patients 70 to 74 years, 20.5% for patients 75 to 79 years, 14% for patients 80 to 84 years, 10.1% for patients 85 to 89 years, and 7.4% for patients aged 90 to 93 years. Black patients were less likely than non-Hispanic White patients to undergo THA (13.9% versus 16.7%). Similarly, patients with low income were less likely than patients without low income to undergo THA (11.5% versus 17.3%). Male patients were slightly more likely than female patients to undergo THA (17.4% versus 16.3%).

The 3 candidate instrumental variables performed well as predictors in the first-stage estimation of surgery type (p < 0.01). The overall volume of THAs at a facility was associated with an increased likelihood of THA (coefficient, 0.0002; p = 0.002), whereas both the proportion of THAs accounted for by fractures (−0.12; p = 0.006) and the proportion of fractures treated with HA (−4.03; p < 0.001) were associated with a decreased likelihood of THA, even after controlling for sociodemographic and clinical factors^28-30^.

### Selection-Adjusted Surgery Type and the Risk of Secondary Hip Surgery and Dislocation at 12 Months

In the fully instrumented and adjusted model (Table III), there was no significant difference in the likelihood of revision/conversion within 12 months postoperatively between patients who received THA and those who received HA (coefficient, −0.008; p = 0.81; adjusted risk of 2.4% and 2.5%, respectively).

**TABLE III:** Surgery Type and 12-Month Outcomes: Multivariable, 2-Stage Instrumental Variable Estimation Results*

	Revision/Conversion			Dislocation		
	Coefficient	Standard Error	P Value	Coefficient	Standard Error	P Value
Surgery type: THA (vs. HA)	−0.008	0.033	0.81	0.17	0.03	<0.001
Other key covariates						
Age, in years						
66-69	Ref.	—	—	Ref.	—	—
70-74	−0.03	0.05	0.52	−0.04	0.05	0.46
75-79	−0.09	0.48	0.068	−0.03	0.05	0.55
80-84	−0.18	0.05	<0.001	−0.11	0.05	0.04
85-89	−0.20	0.05	<0.001	−0.06	0.05	0.25
≥90	−0.33	0.06	<0.001	−0.15	0.06	0.01
Sex						
Female	Ref.	—	—	Ref.	—	—
Male	0.11	0.02	<0.001	−0.01	0.03	0.59
Race/ethnicity						
Non-Hispanic White	Ref.	—	—	Ref.	—	—
Black/African American	−0.25	0.08	0.002	−0.13	0.08	0.09
Hispanic	−0.10	0.07	0.16	0.01	0.07	0.85
Other	−0.23	0.09	0.01	−0.21	0.09	0.02
Low-income status						
No	Ref.	—	—	Ref.	—	—
Yes	−0.045	0.04	0.18	0.09	0.03	0.01
No. of comorbidities	0.02	0.004	<0.001	0.03	0.004	<0.001
Cementation	−0.23	0.03	<0.001	−0.05	0.03	0.07
Overall facility volume of THAs	−0.0003	0.0001	0.02	−0.0004	0.0001	<0.001

* For each model, the dependent variable is a binary indicator given the value of 1 if the patient experienced the outcome (e.g., revision/conversion) and given the value of 0 otherwise. Both models also included a constant term and control for the Census region of the surgery facility, an indicator for unspecified cementation, and residuals from the first-stage (choice of surgery type) instrumental variables estimation. The estimation procedure for each outcome accounted for clustering (i.e., multiple observations within the same facility). Both models were estimated using a probabilistic probit specification to account for the nonlinear, binary nature of the dependent variables and the instrumental-variable assumption of the normality of the residuals in each estimation stage. The regression coefficients represent the adjusted marginal effect associated with a unit change in the indicator covariate, controlling for all other factors. Positive or negative coefficients indicate a higher or lower likelihood, respectively, of experiencing the outcome (e.g., revision/conversion within 12 months after the index arthroplasty surgery). Given the nonlinearity of the dependent variables, the probit regression coefficients do not represent the magnitude of the effect. To provide a sense of the magnitude of the effect of our key variable of interest, surgery type, we utilized these regression coefficients to calculate the predicted (adjusted) probability associated with each surgery type (THA versus HA) for each of the 2 outcomes, while holding all other factors constant at their actual values. These predicted probabilities are presented in the text.

In contrast, patients who underwent THA were significantly more likely than those who underwent HA to experience a dislocation within 12 months (coefficient, 0.17; p < 0.001; adjusted risks of 2.9% and 1.9%, respectively). Despite being small in absolute magnitude, these adjusted risks suggest a 53% greater dislocation rate attributable to the type of surgery.

Relative to the youngest group of patients (66 to 69 years of age [adjusted risk 2.7%]), older age was associated with a lower likelihood of experiencing a revision/conversion within 12 months after the index femoral neck surgery (ages 80 to 84: coefficient, −0.18; p < 0.001 [adjusted risk, 1.8%]; ages 85 to 89: −0.20; p < 0.001 [adjusted risk, 1.5%]; ages ≥90: −0.33; p < 0.001 [adjusted risk, 1.2%]). Similarly, Black/African American patients (coefficient, −0.25; p = 0.002 [adjusted risk, 2.1%]) and patients of a minority race other than Black or Hispanic (−0.23; p = 0.01; [adjusted risk, 1.8%]) were less likely than non-Hispanic White patients (adjusted risk, 3.2%) to undergo revision/conversion. Male patients (coefficient, 0.11; p < 0.001 [adjusted risk, 2.6% for males versus 2.3% for females]) and patients with a higher number of comorbidities (0.02; p < 0.001) were significantly more likely to experience revision/conversion within 12 months. Finally, cementation was associated with a reduced risk of revision/conversion (1.6% with cementation versus 2.8% without cementation; coefficient, −0.23; p < 0.0001).

In addition to THA surgery type, a higher risk of dislocation was associated with factors such as a higher number of comorbidities (coefficient, 0.03; p < 0.001) and low-income status (0.09; p = 0.01 [adjusted risk, 2.8% versus 1.4%]). Patients of non-Black, non-Hispanic minority races were less likely than non-Hispanic White patients to experience a dislocation (coefficient, −0.21; p = 0.02 [adjusted risk, 1.7% versus 2.3%]). Of note, the use of cement trended toward a significant association with a decreased probability of dislocation (−0.05; p = 0.07 [adjusted risk, 1.8% with cementation versus 2.1% without cementation]).

Tests of the adequacy of the instrumental variables indicated that the proportion of the overall facility volume of THAs performed for fracture and the proportion of femoral neck fracture cases treated with HA were valid instruments, as neither was significantly associated with either outcome. However, the total facility volume of THAs remained a significant predictor of outcomes: higher facility case volume reduced the likelihood of dislocation (−0.0004; p < 0.001) and revision/conversion (−0.00028; p = 0.02).

The recomputed first-stage F statistic, in which total facility volume was treated as a covariate rather than an instrumental variable, indicated that the 2 remaining instruments were highly significant (F = 13.7; p < 0.01), demonstrating their validity as instrumental variables^31-33^.

## Discussion

In this nationally representative study of elderly patients undergoing arthroplasty for femoral neck fracture in the U.S., THA was associated with a higher risk of dislocation than HA on instrumental variable analysis, but the overall rates of dislocation were low. There was no significant difference between THA and HA in the adjusted risk of secondary hip arthroplasties (revision or conversion) at 12 months postoperatively. The American Academy of Orthopaedic Surgeons (AAOS) clinical practice guideline describes a possible functional benefit of THA over HA, but at the risk of increased complications^34^; our data are consistent with the reported increased in complication risk as it pertains to dislocation.

The clinical decision to choose HA or THA is multifactorial. Relevant factors include the potential for improved functional outcomes with THA and the potential for lower complication risk with HA. In its clinical practice guideline, the AAOS advocates for the “discussion of risk and benefit with patients and families” in the form of “shared decision making.”^34^ Our results further inform the discussion of risks with precise estimates of the risk of dislocation and revision in a U.S. population. Additionally, our data revealed significant differences in outcomes by age, sex, race, income status, and cement status. We found that advanced age (≥80 years), Black or “other” minority race/ethnicity, female sex, a lower number of comorbidities, and the use of cement were protective against revision/conversion. Additionally, advanced age (80 to 84 years or ≥90 years), “other” race/ethnicity, non-low-income status, and a lower number of comorbidities were protective against dislocation, with use of cement trending toward a significant association with a lower risk of dislocation.

Previous studies have shown conflicting results regarding the comparative revision risks associated with THAs and HAs performed for the treatment of femoral neck fracture. Some relatively small randomized trials^8-13^ have shown improved implant survival with THA as compared with HA, but these results have not been consistently reproduced in other randomized trials^14-17^. Notably, a large, international RCT showed no difference in revision rates between the procedures at 2 years postoperatively but did demonstrate a nonsignificant trend toward higher revision rates at 12 months following THA^16^. Meta-analyses of RCTs have shown a lower revision risk at long-term follow-up^31,32^ but higher rates of early revision^31^ following THA. A population-based analysis from Canada found no difference in revision rates between the procedures at early or long-term follow-up^18^.

Given the uncertainty regarding early revision and the lack of data from population-based data sets in the U.S., we conducted an analysis focused on the risk of revisions/conversions at 12 months following the index arthroplasty with use of a large Medicare data set. Our results showed that THA was not associated with an increased risk of revision/conversion at 12 months. Revisions for acetabular erosion, a known long-term complication of HA, were not reflected in our data.

Historical data have shown a higher risk of dislocation associated with THA, with 3 RCTs from 1986, 1989, and 2000 demonstrating dislocation rates of 12% to 20%^33,35,36^. Meta-analyses that included these studies showed an increased risk of dislocation following THA versus HA^31,32,37^. More recent RCTs showed dislocation rates between 0% to 5% following THAs performed for femoral neck fracture^15,16^, and an updated meta-analysis showed no difference in dislocation rates between the 2 procedures^38^.

Our data showed a significant difference in dislocation risk between the HA and THA groups, with a dislocation risk of 2.9% following THA and 1.9% following HA. These risks of dislocation are consistent with data from the more recent RCTs showing rates of <5%. Similarly, 2 population-based studies from Canada found dislocation rates of <2% for THA and HA in the treatment of femoral neck fracture^18,39^. Both of these studies demonstrated that THA was associated with an increased risk of dislocation despite the low rates—similar to our own findings^18,39^. In contrast, a population-based analysis from France, in which dual-mobility implants were utilized in 18% of the procedures in the THA group, demonstrated that THA was associated with a reduced risk of dislocation, despite having found a dislocation rate of 5.9% in the THA group^19^. We utilized dislocation and not revision for dislocation as an end point because only a minority of patients with dislocation following arthroplasty for femoral neck fracture undergo revision surgery^40,41^.

The limitations of the present study include the use of observational data. It is possible that unobserved variables biased the results, but we attempted to limit this with use of an instrumental variable analysis, as has been done previously in studies of THA versus HA for femoral neck fracture^25^. Second, we were unable to control for surgical approach, implant details including the femoral head size or the use of dual mobility, and body mass index, which may have influenced the rates of dislocation and revision/conversion. Third, we did not report data regarding mortality, quality-of-life outcomes, long-term results, or costs, which are important factors in surgical decision-making. Fourth, we were unable to report the reasons for conversion/revision. Lastly, the majority of the patients in this analysis were non-Hispanic White, which may limit the generalizability of the results; we utilized all of the available cases in the Medicare data set, and thus the racial composition of our cohort reflects the data that were available in the data set.

### Conclusions

The findings from this selection-corrected analysis of the type of surgery (HA versus THA) on outcomes demonstrated that the treatment of displaced femoral neck fractures with THA in elderly patients was associated with a significantly increased risk of dislocation at 12 months postoperatively, although the difference may not be clinically important. The overall rate of dislocation was low in both the HA and THA groups. The risk of revision/conversion at 12 months postoperatively did not differ between the groups.
